# MicroRNA-433-3p promotes osteoblast differentiation through targeting DKK1 expression

**DOI:** 10.1371/journal.pone.0179860

**Published:** 2017-06-19

**Authors:** Xiaolin Tang, Jiantao Lin, Guanhai Wang, Jianlin Lu

**Affiliations:** 1 Department of Medical Science, Shunde Polytechnic, Foshan, China; 2 Traditional Chinese Medicine and New Drug Research Institute, Guangdong Medical University, Dongguan, China; Università degli Studi della Campania "Luigi Vanvitelli", ITALY

## Abstract

Dickkopf-1 (DKK1) is a powerful antagonist of canonical WNT signaling pathway, and is regarded as a biomarker for osteoporosis. Its expression is highly correlated with bone mass and osteoblasts maturation. In this study, mouse primary bone marrow cells and osteoblast cell lines were used. Luciferase reporter assay and western blotting methods were employed to validate if miRNA-433-3p epigenetically regulated DKK1 translation. Rat bone marrow derived osteoblasts were infected with lentivirus vector in which miR-433-3p was constructed. The authors constructed lentivirus mediated miRNA-433-3p stable expression and examined the alkaline phosphatase (ALP) activity and mineral deposition level in vitro. In situ hybridization method was used to observe miR-433-3p in primary osteoblasts. We built up an OVX rat model to mimic postmenopausal osteoporosis, and found aberrant circulating miR-433-3p and miR-106b, which were not reported previously. Results showed that miR-433-3p potentially regulated DKK1 mRNA, Furthermore, the correlation of serum DKK1 with circulating miR-433-3p level was significant (r = 0.7520, p = 0.046). In the luciferase reporter assay, we found that miR-433-3p siRNA decreased luminescence signal, indicating direct regulation of miR-433-3p on DKK1 mRNA. When the miR-433-3p binding site in DKK1 3’UTR was mutant, such reduction was prohibited. Western blotting result validated that miR-433-3p inhibited over 90% of DKK1 protein expression. Similarly, the change of protein expression was not observed in mutant group. The stable expression of lentivirus mediated miR-433-3p increased ALP activity and mineralization both in human and rat derived immortalized cells. We found that primary osteoblasts had higher miR-433-3p level compared with immortal cells through real-time PCR, as well as in situ hybridization experiment. Conclusively, our findings further emphasized the vital role of miR-433-3p in DKK1/WNT/β-catenin pathway through decreasing DKK1 expression and inducing osteoblasts differentiation.

## Introduction

The canonical Wnt/β-catenin signaling pathway activates bone formation and resorption genes transcription. It is a vital factor in regulating osteoblast differentiation, proliferation, survival, and ultimately bone formation[1]. Dickkopf-1 (DKK1) is a soluble potent antagonist of canonical WNT protein. DKK1 blocks WNT/β-catenin signal pathway by binding to Wnt co-receptor LRP5/6 and Kremen 1/2, thereby sequestering LRP5/6 from the trans-membrane Frizzled receptor[2]. Overexpressing DKK1 in osteoblasts was found to decreased osteoblast numbers and in osteopenia[3], whereas DKK1 allele single deletion was associated with increased bone formation and bone mass in another murine model[4]. Many studies reported that blocking the function of Dkk-1 benefited bone maintenance and protected from systemic bone loss[5, 6]. Current opinions on osteoporosis realized that DKK1 level was associated with the pathophysiology of postmenopausal osteoporosis[7, 8], and with the inflammatory cytokines effects on bone mass[9, 10]. Ahmed et al. showed postmenopausal women with significantly increased serum Dkk-1 had more severe osteoporosis, indicating that higher level of serum Dkk-1 might act as a biomarker for the development and severity of osteoporosis[7]. In this study, we mainly focused on the postmenopausal osteoporosis.

Osteoporosis is a bone metabolic disorder disease, which is related with hormone secretion, age, bone metabolic, and chronic inflammatory diseases[11]. Bone homeostasis is precisely controlled by osteoblasts and osteoclasts[12]. Osteoporosis happens when there is excessive bone resorption and/or reduced bone formation. Therefore, cell-cell direct and indirect communication, or feedback loop between osteoblasts and osteoclasts may play an important role in bone metabolism[13, 14]. The signal mediator could be small chemicals, peptides, proteins, and even microRNAs[15–17]. Recent studies found that the exosomes or called extracellular vesicles secreted by bone-related cells played important roles in bone homeostasis[13, 18]. These small exosomes are mainly 10 nm to 200 nm in diameter large, bilayer liposome structure, secreted through a paracrine or endocrine manner to facilitate a diversity of intracellular or intercellular signaling mechanisms. Once exosomes are released, they can either target a neighboring cell or reach cells of distant organs after entering the blood stream[19]. However, the exact mechanisms of regulation on target cells and feedback signal by exosomes are poorly understood. Discovery of contents in exosomes and their target cells might uncover the mechanism of cell-cell communication during osteoporosis development.

microRNAs (miRNAs, miRs) regulate multiple processes in bone homeostasis, including osteoblast and osteoclast differentiation, orchestration of bone programming and management of cell fate[12]. Circulating miRNAs were reported to act as cell fate determining factors within cell-secreted exosomes. These circulating miRNAs were protected from RNase degradation because they were capsulated within bilayer-lipid exosomes[15, 20]. These miRNA-containing exosomes precisely delivered contents to target cells through ligand-receptor interaction method. It implied the ability of miRNAs of influencing the physiological behavior of recipient cells via circulation[21]. The present study aimed to uncover the circulating miRNAs and their effects on osteoporosis development, as well as their origins. We mainly focused on those aberrant circulating miRNAs which were discrepant ovariectomized (OVX) rat from sham rat in vivo.

## Materials and methods

### 1. Ovariectomized rat as osteoporotic model and statement

This study was carried out strictly accordance with the recommendations in the Guide for the Care and Use of Laboratory Animals of the National Institutes of Health. The study and protocol were approved by the Animal Care and Use Committee of Guangdong Medical University(Permit Number: GMDU-16-01651). Ten female Sprague-Dawley (SD) rats were provided and raised by Animal center of Guangdong Medical University (Zhanjiang, China). The rats were housed in a standard animal facility under controlled temperature (22°C), and 12-h light dark cycle, with free access to fresh water and low-calcium food pellets. Following the FDA guidelines on rat OVX model[22], the OVX model for osteoporosis was generally applied for research related to postmenopausal osteoporosis, showing trabecular bone BMD loss and significant reductions in biomechanical strength after OVX[23]. Rat ovariectomy surgery was performed under anesthesia and analgesia, and all efforts were made to minimize suffering. Six rats were ovariectomized as model group, and four rats as sham group. The protocol followed Cui's published study recently[24]. Briefly, six-month old rat was anaesthetized by injection of ketamine (100 mg/ml, Sigma-aldrich, MO) and xylazine (10 mg/ml, Sigma-aldrich,) mixture 0.1 ml/100g through i.p. In model group, rat bilateral fallopian tubes were knotted by surgical suture for occlusion. In sham group, only surgical operation was performed and simply touched the fallopian with forceps without fallopian occlusion. After operation, rats were kept warm by lighting until awaked. If the rat showed a pain suffering symptom and did not relieve after 6 h, injected xylazine 0.5 mg/100g body weight. Animal daily care after ovariectomy operation followed ordinary protocol, and did not receive special care. Rats were monitored every day, and observed their behavior and live status. If a rat was severe weak and did not reach the osteoporotic disease model, the rat would received euthanasia by standard protocol. After 3 months post ovariectomy, rat osteoporotic model was built up. Rat euthanasia method used injection of 3 times of anesthetics chemical dose, which was 0.3 ml/100g of ketamine and xylazine mixture.

### 2. Cell culture

Human hFOB1.19 (ATCC, #CRL-11372)was purchased from American Type Culture Collection. The culture medium for hFOB 1.19 cell is a 1:1 mixture of Ham's F12 Medium and Dulbecco's Modified Eagle's Medium, supplemented with 2.5 mM L-glutamine (without phenol red), 0.3 mg/ml G418 and fetal bovine serum to a final concentration of 10%. Human hFOB1.19 cells were cultured in incubator with temperature 34°C, 100% humidity and 5% CO2. Rat ROS17/2.8 cell line was kept and stored in our department, which was developed by Dr. Gideon Rodan. They were cultured with α-MEM medium with 5% FBS and 1 nM dexamethasone. ROS 17/2.8 cells were cultured with DMEM/Ham's F-12 medium (1:1) (Gibco, Thermo) containing 10% inactivated fetal bovine serum (Gibco, Thermo), supplemented with 2 mM Glutamine, 100 Units Penicillin/mL and 100 μg streptomycin/mL (Sigma-aldrich, MO), at 37°C in a humidified atmosphere of 5% CO2. Primary bone marrow-derived mesenchymalsstem cells (MSCs) were isolated from 4-month old SD female rat. Primary cells were cultured in OIM medium supplemented with 1 nM dexamethasone, 50 μM ascorbic acid, and 20 mM β-glycerolphosphate. Then cells were proliferated with α-MEM medium supplemented with 10% FBS, 100 U/ml penicillin, 100 mg/ml streptomycin, and 2 mM L-glutamine (Thermo) and cultured at 37°C, 5% CO2.

### 3. Luciferase reporter assay

mRNA and miRNA target prediction were used online software: http://www.targetscan.org/vert_71/ and http://www.microrna.org/microrna/searchMirnas.do. In the following validation, full length 3'UTR of DKK1 mRNA (Accession No.: NM_012242) was constructed into pmirGLO vector MCS cloning site (Promega). Nucleotide synthesis was finished by Genscript Inc. MiR-433-3p siRNA oligonucleotide was purchased from Santa Cruz Biotechnology. Blank or constructed vectors were co-transfected with miR-433-3p oligonucleotide into cells using Fugene HD lipid (Roche, # 04709705001). After 24 h of transfection, we performed luciferase reporter assay experiment, following manufactory's protocol which was provided by Promega company (#E1910). Luminescence signal of luciferin was detected by BioTek Synergy 2 machine (BioTek Inc.). Briefly, we removed the growth medium from cultured cells, and rinsed cultured cells in 1×PBS. Then, we removed all rinse solution and dispensed recommended volume (for 48 well plate) of 1×PLB buffer into each culture vessel. The culture vessel was gently shook for 15 minutes at room temperature. Then, we transfered 65μl of PLB Lysate into each well, and mixed well. Firefly luciferase activity were then measured and recorded by Synergy 2 plate reader. After dispensed 100μl of Stop & Glo^®^ Reagent, we measured renilla luciferase activity and recorded again as reference signal. The firefly luciferase signals were compared between different treatments before referred to renilla luciferase signal.

### 4. real-time PCR

Total mRNA was extracted by TRIzol reagent (Thermo Fisher Scientific inc.). We followed the extraction procedures provided by the manufactory. Briefly, we removed growth media, and then added 0.3–0.4 mL of TRIzol^™^ Reagent per 1×10^6^ cells directly to the culture dish to lyse the cells. Cell lysate was pipetted up and down several times to homogenize.0.2 mL chloroform was added per 1 mL of TRIzol^™^ to the mixture. After centrifuge the sample for 15 min at 12,000g at 4°C, the aqueous RNA phase was transfered to a clean tube, and precipitated with 100% isopropanol. The sample was centrifuge for 10 min at 12,000g at 4°C. The RNA pellet was washed with 75% ethanol, and centrifuged at 12,000g at 4°C for 10 min. The RNA pellet was air-dry for 10 min, and dissolved with 20 μl RNase-free water. Totally 1 μg of extracted RNA were reverse-transcribed into cDNA using miRNA reverse primer[25]. Real-time PCR primers for circulating miRNAs were listed in supplemental S1 Table. U6 RNA and its primers as control. PCR was carried out at 94°C for 30s, 55°C for 30s and 72°C for 45s for 30 cycles, using ABI prism 7700 sequence detector and software to analyze the data (Applied Biosystems, CA).

### 5. Alizarin red staining

Differentiated osteoblasts could be induced to produce vast extracellular calcium deposits in vitro, which is called mineralization. The deposited calcium deposits could be specifically stained bright orange-red using Alizarin Red S. In the mineralization study, rat bone marrow derived cells were plated at density of 5×10^3^ cells/cm^2^ in a 12-well plate and cultured with growth medium until the cells reached 80% confluence. After that, cells were cultured with osteogenic induction media (OIM), which were supplemented with 1 nM dexamethasone, 50 μM ascorbic acid, and 20 mM β-glycerophosphate (Sigma-Aldrich, MO). Cells were placed in 37°C and 5% CO_2_ incubator (#150Li, Thermo). After seven days induction, we measured mineralization of osteogenic induction using alizarin red staining method. The plate was washed with PBS three time, and fixed with 70% ethanol for 30 min. After fixation, 1 ml/well of 0.5% Alizarin red S (Sigma-Aldrich, MO) was added to each well. The volume should cover the cellular monolayer. Plate was incubated at room temperature in the dark for 45 min. Then carefully aspirated the Alizarin Red S solution and washed the cell monolayer four times with PBS. Mineralized osteoblasts were stained in bright orange-red because extracellular calcium deposits, whereas undifferentiated cells were very slightly red or colorless. The plate was viewed by Epson scanner and recorded.

### 6. Western blotting

Cultured osteoblasts were washed with ice-cold PBS three times, and lysed with ice-cold lysis buffer, supplemented with protease and phosphatase inhibitors. After centrifugation at 12, 000 g 4°C for 15 min, supernatant was collected and protein concentration was quantified. We made 12% separating SDS gel for DKK-1, and 10% gel for actin separation, 5% stacking gel for both proteins. Twenty microgram of total protein were loaded into each lane. In protein SDS-PAGE electrophoresis, 110 V running votage was set from the beginning to the finishing of electrophoresis. The proteins were transfered onto Hybond C nitrocellulose membrane using blotting cassette(GE Healthcare, Arlington Heights, IL) with 100 v in ice-cold condition for 60 min. The membrane was blocked with 5% no-fat milk TBST at room temperature for 30 min. After blocking, membrane was incubated with primary antibodies (1:2000 dilution in TBST) over night at 4°C with slightly shaking. After 4 times of TBST wash, the membrane was probed with horseradish peroxidase-conjugated secondary antibodies (1:3000 dilution in TBST) for another 1 h at room temperature. Chemiluminescent signals were then developed with ECL reagent (Healthcare Inc.) and detected by the ChemiDoc XRS gel documentation system (Bio-Rad).

### 7. ALP staining

Differentiated osteoblasts showed ALP activity, which was greatly enhanced during bone formation. ALP activity was therefore a feasible marker for osteoblast differentiation. ALP could easily be detected using BCIP/NBT as a substrate, which stained cells blue-violet when ALP was present. In the beginning, bone was fixed and decalcified according to published reference with slight modification [26]. 8% formal nitric acid in 10% paraformaldehyde solution was used to decalcify and fix rat femur for three days. Decalcified bone was sectioned at 5 μM with microtome (Leica). Bone osteoblasts were stained using ALP staining kit (CosmoBio, Japan). Staining protocol followed manufactory's protocol with modification. Briefly, decalcified sections were de-waxed and cleared with gradient ethanol 100%, 95%, 75% and 50%, each gradient for up-down washing for 5 min. Sections were washed with distilled H_2_O finally. Clear sections was washed with PBS twice, followed by adding 50uL of chromogenic substrates to each section, and incubated at 37°C for 20–60 minutes. When cells staining of ALP were clearly visualized, we stopped the reaction by adding excess deionized water. ALP-positive cells containing ≥3 nuclei were counted under a light microscope.

### 8. Digoxigenin (DIG)-labeled miRNA probe in situ hybridization

In situ hybridization experiment, we followed standard protocol provided by miRNA probe manufactory (Focofish, Guangzhou). We synthesize the anti-miRNA sequence for subsequent DIG labeling (Genscript Inc). The probe sequences for human was 5'-ACACCGAGGAGCCCATCATGAT-3'. Locked nucleic acid-based miR-433-3p probe was labeled with DIG at the 3' end (Focofish, Guangzhou). U6 snRNA was used as positive control. Decalcified sections was prepared previously and ready to use. After being washed with PBS, sections were treated with 24 mM HCl in ethanol and 20 μg/ml proteinase K for 10 min to reduce endogenous and non-specific binding and fluorescence. Sections were incubated with miR-433-3p probe in a moist chamber at 50°C overnight. Slides were washed with PBST for 3 times with horizontal shaking for 5 min. Subsequently, slides were placed in a dark box, and blocked with 2% BSA in PBST for 30 min. Sections were incubated with anti-DIG secondary antibody labeled with Rhodamine B. Sections were incubated with DAPI dye for 10 min at room temperature. Then anti-fade reagent was added onto each section to avoid fluorescence fading before mounting with glycerol and cover slide. Slides were visualized under fluorescent microscopy (Leica, Germany).

### 9. Statistics

All statistic analysis were performed using the Statistics Package for Social Sciences (SPSS for Windows, version 13.0, SPSS Inc, Chicago, IL). U-test was used for comparison of continuous variables between two groups if sample size was small. For more than two groups comparison, ANOVA test was used. P value < 0.05 was deemed as significantly different.

## Results

### 1. Circulating miRNAs indicated for development of osteoporosis in ovariectomized rat

In the osteoporotic animal model building procedures, ovariectomized (OVX) rat and subsequent drug treatment were administrated to reach the criteria of osteoporosis. The standard protocol was followed Lin's paper published previously[24]. After building up the OVX rat model, selected serum miRNAs were quantified through real-time PCR method (primers shown in supplemental S1 Table). Real time PCR results of selected miRNAs were listed in Table 1. We also compared the results with peers' studies on OVX rat or postmenopausal women, which were published very recently. Our findings were coincident with most previous studies on human in circulating miRNAs quantification, but still there were some new findings. We found miR-433-3p and miR-106b were not reported previously in OVX rat circulating serum. Taken together, we successfully built up an OVX rat model to mimic postmenopausal osteoporosis, and found some interesting circulating miRNAs which might played an important role in the development of osteoporosis.

**Table 1:** Summary of selected miRNAs and serum quantification in rat model.

miRNAs	Expression level/sham group (-2^ΔΔCT^)	Status (vs. sham rat)	Peers' studies
hsa-miR-34a-3p	2.63	up	Up, Jess Morhayim[15]
hsa-miR-433-3p	1.24	up	This study
hsa-miR-106b	2.24	up	This study
hsa-miR-23a	0.48	down	Down, Sylvia Weilner[27]
hsa-miR-328-3p	0.38	down	Down, Sylvia Weilner
hsa-miR-29b-3p	2.1	up	Up, Jess Morhayim
hsa-miR-146a-5p	2.68	up	Up, Jess Morhayim
hsa-miR-148a-3p	1.85	up	Up, Cheng[28]

### 2. miRNA-433-3p targeted DKK1 protein expression

**Fig 1:**
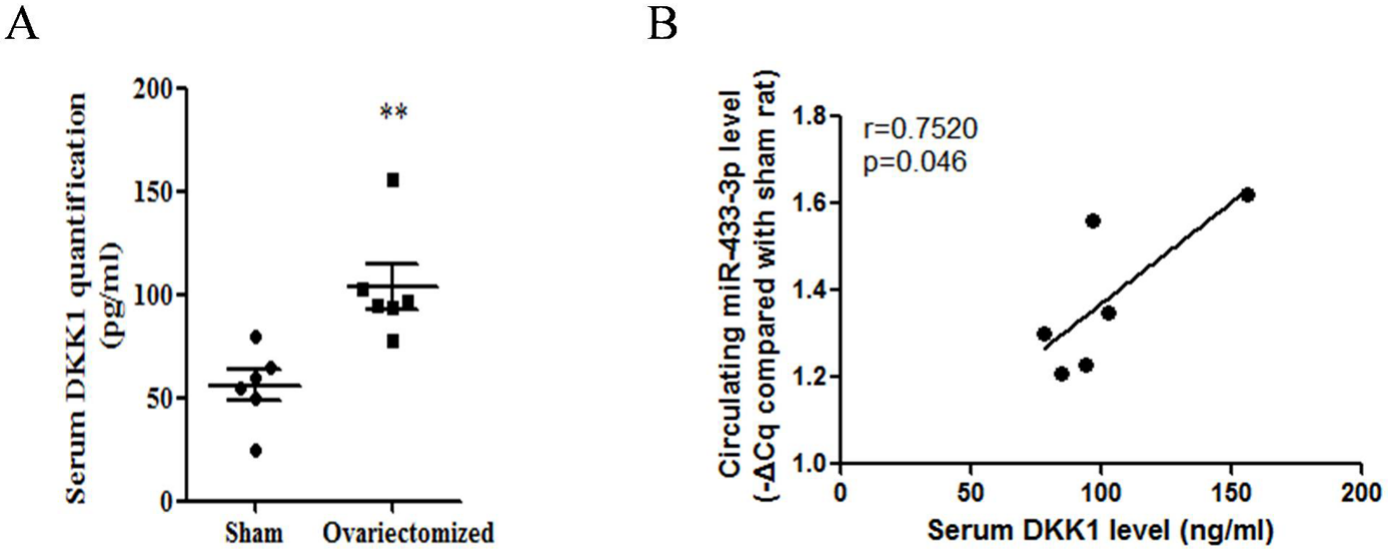
Correlation of serum DKK1 with circulating miR-433-3p. Up-regulated DKK1 level was found in osteoporotic rats compared to sham control rats (A, n = 6, U-test, p<0.01). Correlation of serum DKK1 with circulating miR-433-3p (B, linear regression, r = 0.7520, p = 0.046).

We noted that DKK1 played important role in the development of osteoporosis. Thus it was essential to determine if serum level of DKK1 was association with circulating miRNA Firstly, we quantified the DKK1 protein level in rat serum through commercial ELISA kit (#DKK100, R&D Systems.). All procedures followed manufactory's instruction. Results showed increased up-regulated DKK1 level was found in osteoporotic rats compared to sham control rats (Fig 1A, n = 6, p<0.01). To explore whether DKK1-targeting miRNAs level was changed because of the pathogenesis of osteoporosis, we predicted miRNA targets through online software, and analyzed their correlation association. We predicted the potential miRNAs which would regulate DKK1 express using online software and database (http://www.targetscan.org/vert_71/). Data showed that miR-433-3p potentially regulated DKK1 mRNA. Importantly, miR-433-3p was increased in serum and was contained in osteoblast secreted exosomes which targeted umbilical cord blood cells[15]. Furthermore, the correlation of serum DKK1 with circulating miR-433-3p level was analyzed by SPSS software. Result indicated that their correlation was significant, and moderate association, r = 0.7520, p = 0.046 (Fig 1B).

**Fig 2:**
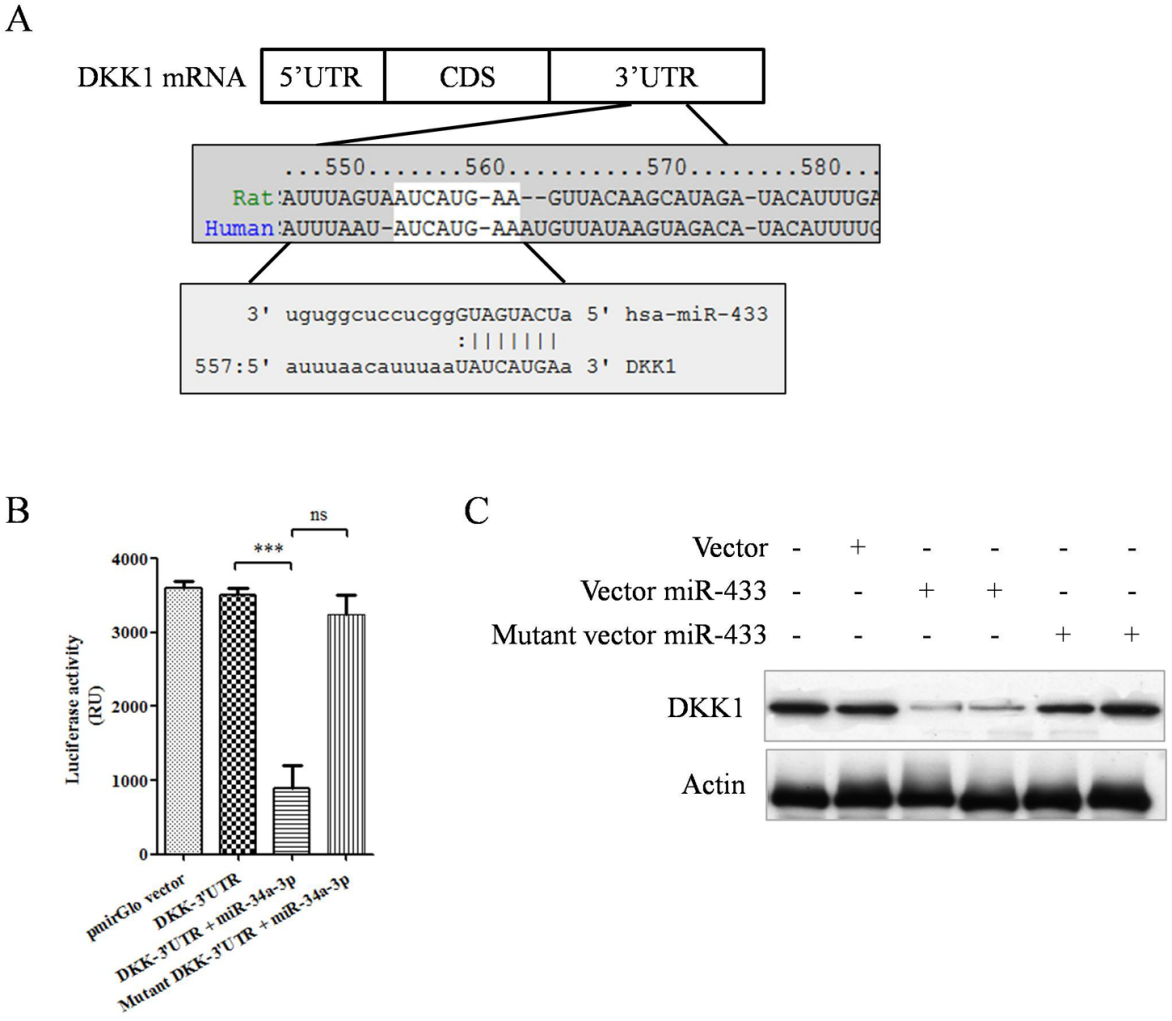
Validation of miR-433-3p targeting DKK1 mRNA 3’UTR. Schematic figure showed miR-433-3p targeting DKK1 mRNA 3'UTR, mapping from site 554 to 560 (A). In the luciferase reporter assay, data showed vector constructed DKK1 mRNA 3'UTR group had quite lower luminescence signal in the present of miR-433-3p siRNA co-transfection simultaneously, whereas binding site mutant group had no reduction in luminescence signal (B, ANOVA, p<0.001, Mean ± SE of triple experiments with four duplicate wells). Protein immunoblotting assay showed miR-433-3p inhibited DKK1 expression up to 90% of total protein (C).

Therefore, we hypothesized that these exosomes might target osteoclasts to regulated DKK1 level. Schematic figure of binding sequence and site was shown in Fig 2A. In the luciferase reporter assay, results showed vector constructed with DKK1 mRNA 3'UTR had lower luminescence signal in the present of miR-433-3p siRNA oligonucleotide transfection simultaneously (Fig 2B, ANOVA, p<0.001, 4 replicate wells each group, error bars shown as SE of triple experiments). When the miR-433-3p binding site in 3’UTR was mutant, such reduction was prohibited (Fig 2B, column 4). To further validate the regulation of miR-433-3p on DKK1 expression level, protein immunoblotting assay showed that increased miR-433-3p inhibited over 90% DKK1 protein expression (Fig 2C, p<0.0001 compared to control group). Significant change of DKK1 protein expression was not observed in mutant group.

### 3. Administration of miRNA-433-3p promoted osteoblasts differentiation in vitro

**Fig 3:**
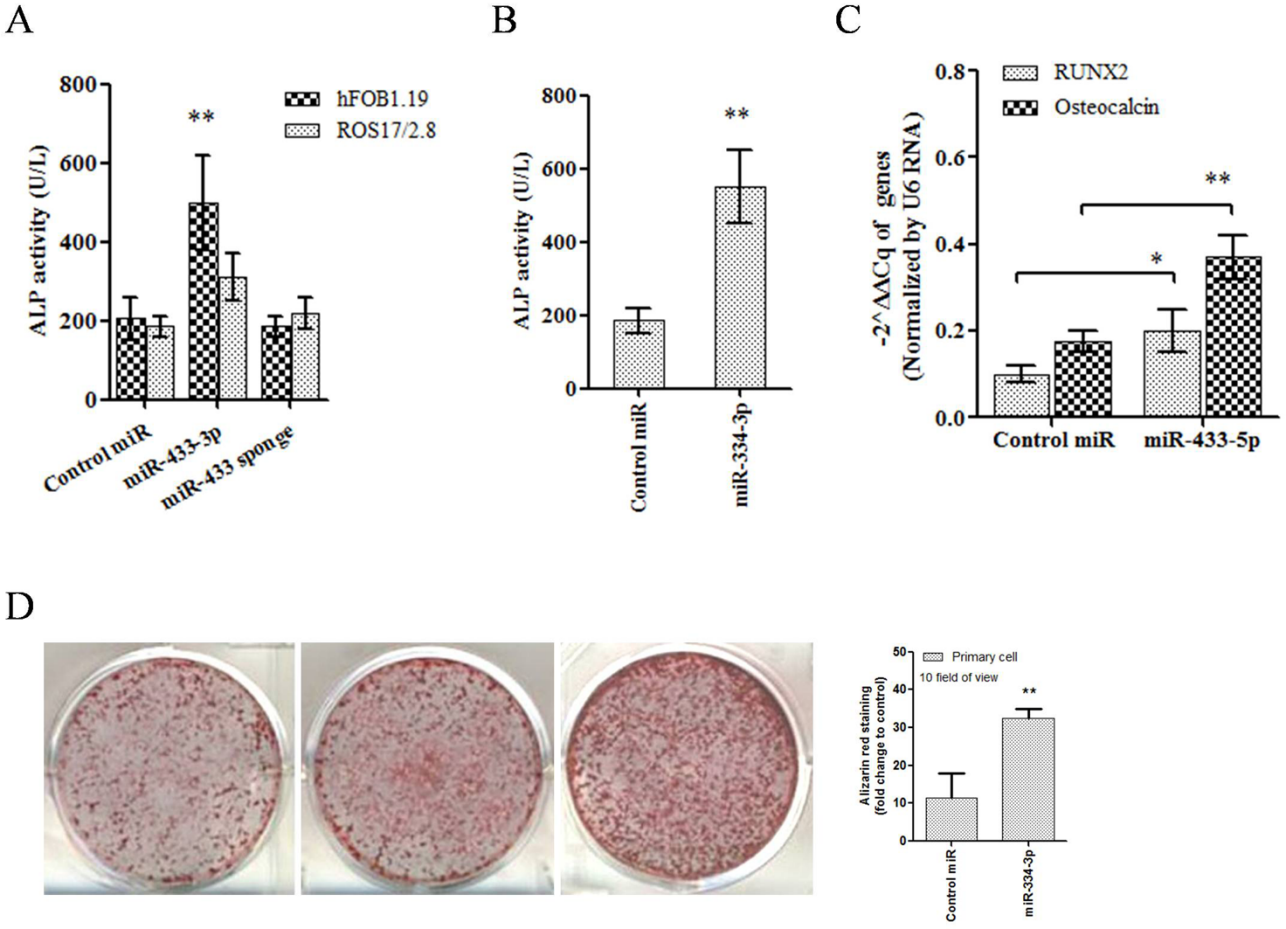
Effects of miR-433-3p on osteoblasts differentiation and maturation. Lentivirus mediated miR-433-3p stable expression increased ALP activity of immortalized human hFOB1.19 and rat ROS 17/2.8 cells (A, p<0.01, triple experiments). miR-433-3p also increased primary bone marrow induced ostetoblasts ALP activity, but not in miR-433-3p plus sponge vector group (B, **, p<0.01). Infection of lentivirus miR-433-3p to human hFOB1.19 cells showed increased mRNA level of osteogenic markers RUNX2 and osteocalcin (C, **, p<0.01). Alizarin red staining results indicated that miR-433-3p promoted Ca^2+^ deposited within osteoblasts (D, p<0.01). Bar chart showed ten counting of views under microscopy of each group.

We have shown the direct regulation of miR-433-3p on DKK1, but the effect of miR-433-3p on osteoblasts was still not clear. To answer this question, we performed ALP activity assay and mineral deposition assay in osteoblast cell lines and primary bone marrow-derived osteoblasts. Cells were infected with lentivirus in which miR-433-3p sequence was constructed (plenty 7.3/V5-DEST). Results showed that lentivirus mediated miR-433-3p stable expression increased ALP activity both in human and rat derived immortalized cells compared to controls (Fig 3A, mean ± SE of triple experiments, p<0.01). Increased ALP activity was not detected in miR-433 sponge vector group. Rat bone marrow derived osteoblasts were infected with miR-433-3p stable expression lentivirus. After 7 days infection, ALP activity of primary osteoblasts was measured. Results showed that lentivirus mediated miR-433-3p stable expression also increased osteoblasts ALP activity compared to blank lentivirus (Fig 3B, mean ± SE, p<0.01). Mineral deposition assay was used to show the differentiation and maturation of osteoblasts. Alizarin red staining figures indicated that miR-433-3p promoted Ca^2+^ deposited within osteoblasts (Fig 3C, under microscopy counting for ten views, mean ± SE, p<0.01).

### 4. Osteoblasts harbored high level of miRNA-433-3p

**Fig 4:**
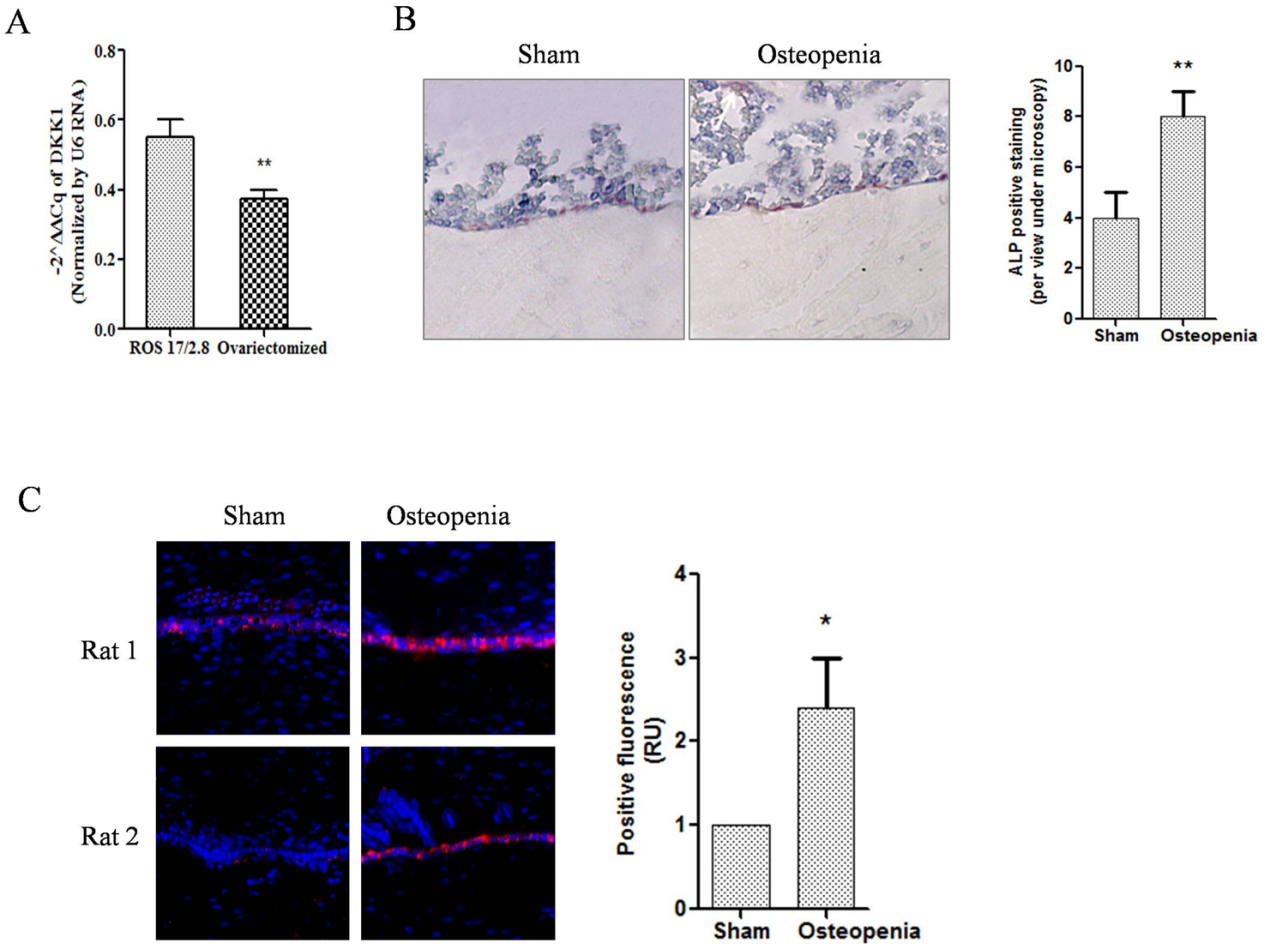
miRNA-433-3p quantification and staining in bone tissue. Data shown primary cultured osteoblasts had higher miR-433-3p level compared with immortal cells using real-time PCR(A, p< 0.01). Red cells showed osteoblasts by ALP staining in decalcified bone tissues (B). DIG-labeled antisense of miR-433-3p probe was detected by anti-DIG secondary antibody, thus red color was observed under microscopy with 510–530 nm excitation (C, Rhodamine labeled, p<0.05).

In order to elucidate if primary osteoblasts harbor high level of miR-433-3p, we quantified the miR-433-3p in cells using real-time PCR. Results showed that primary osteoblasts had higher miR-433-3p level compared with immortal cells (Fig 4A, p< 0.01). We further analyzed the miR-433-3p level in bone tissue of rat femur bone using ALP staining method and in situ hybridization method. Firstly, femur bone was fixed and decalcified in the beginning as described previously. Femur bone osteoblast ALP staining was visualize and confirmed under microscopy. (Fig 4B, **, p<0.01, red cells showing osteoblasts). In the experiment of in situ hybridization, nucleus were stained by DAPI dye, and emitted blue light through UV excitation. miR-433-3p oligonucleotide was stained by rhodamine B, which would emit red color through excitation. Results from in situ hybridization data showed that high level of miR-433-3p was found in femur bone osteoblasts (Fig 4C, p<0.05, ten countings of microscopy views).

## Discussion

Bone metabolic signal and homeostasis which were triggered by osteogenic cells would play even important roles during the development of osteoporosis. Circulating miRNAs have been identified as biomarkers for a variety of diseases, including in osteoporosis[29], cancer[30]and metabolic related diseases[31]. Specifically, a number of very recent studies had identified the differences in tissue and circulating miRNAs in the serum of osteoporosis. Studies showed that circulating miRNAs could be taken up by cells and thereby influence the recipient cell's behavior in the context of diverse biological functions[32, 33]. Enlighten by those findings, we designed this study to identify significant circulating miRNAs in osteoporosis via OVX model. In the present study, aberrant circulating miRNAs were found in osteopenia rat model, confirming the important roles of miRNAs in diagnosis and therapy for osteoporosis. In agreement with Jess Morhayim, Sylvia Weilner and Cheng' studies[15, 27, 28], we showed miR-433-3p and miR-106b were up-regulated in OVX rat model. Previously, peers' studies have demonstrated the association of increased serum DKK1 level in postmenopausal osteoporosis and decreased bone mass[7, 8]. We further showed the association of serum DKK1 and circulating miR-433-3p (r = 0.7520, p = 0.046). Importantly, this study linked the association of high level of DKK1 and miR-433-3p in OVX rat. These findings indicated a new insight of feedback loop between osteoblast and osteoclast through circulating miRNAs and WNT pathway regulator.

In the design of this study, we hypothesized that high level of miR-433-3p in osteoblasts might be associated with the negative feedback of high DKK1 level in serum. We thought that was the natural function of osteoblast to against osteoclast regulation network. As far as we know, osteoblasts regulated osteoclasts mainly through receptor activator for nuclear factor-κB ligand (RANKL) and osteoprotegerin (OPG) signals[34, 35], EPHB4-EFNB2-mediated switch signal[36] and Sema4D-Plexin-B1-RhoA signal[37]. Osteoclasts modulated osteoblast via transforming growth factor-β, sphingosine-1-phosphate, ephrins and semaphorins[38]. Studies reported that circulating signature of miRNAs in plasma or serum correlated with presence and progression of osteoporosis pathological conditions[29, 39]. Circulating miRNAs were protected from RNase degradation because of dilayer-lipid membrane. These dilayer-lipid membrane exosomes contained miRNAs, proteins, and other intracellular substance. The recent studies showed that exosomes which were secreted by osteoblasts targeted osteoclasts through ligand-receptor recognition way[15, 27]. In another aspect, osteoclasts could also secret exosomes to modulate osteoblast function. Sun et al. provided evidences and testified that osteoclasts secreted circulating miR-214 to inhibit osteoblast activity[18]. In agreement with the above findings, we showed that osteoblasts harbored high level of miR-433-3p (Fig 3) which might secret to circulating or tissue space. Results further validated our hypothesis that osteoblasts could modulate osteogenic bone formation cells via secreting exosomes. This finding was in agreement with previous studies[15, 27, 28].

Furthermore, we validated that miR-433-3p directly regulated DKK1 expression through epigenetic modulation mechanism. When human hFOB1.19 cells were infected with recombinant lentivirus which stably expressed miR-433-3p, results showed increased mRNA level of osteogenic markers RUNX2 and osteocalcin. The RUNX2 and osteocalcin were reported negatively associated with DKK1 level in serum and in osteoclasts[40–42]. Alizarin red staining results indicated that miR-433-3p promoted Ca2+ deposited within osteoblasts. As no study reported DKK1 regulated by miR-433-3p, we further confirmed such regulation by mutant miR-433-3p method. Data showed mutant miR-433-3p lost the regulation ability on DKK1 expression. We also used miRNA sponge technology to silence miR-433-3p level, and found that miRNA sponge decrease ALP activity in the present of miR-433-3p in hFOB1.19 and ROS 17/2.8 cells. However, we don't have direct evidences to show miR-433-3p was secreted by osteoblasts and targeted osteoclasts to reduce DKK-1 sercretion via exosomes.

Conclusively, the present study confirmed the concept that osteoblast could modulate osteogenic bone formation network via secreting exosomes. The authors further emphasized the vital role of miR-433-3p in DKK1/WNT/β-catenin pathway through decreasing DKK1 expression and inducing osteoblasts differentiation.

## Supporting information

S1 Table Primers for reverse transcription and real-time of miRNA. (XLSX)
